# Comparison of serum and urinary biomarker panels with albumin/creatinine ratio in the prediction of renal function decline in type 1 diabetes

**DOI:** 10.1007/s00125-019-05081-8

**Published:** 2020-01-08

**Authors:** Marco Colombo, Stuart J. McGurnaghan, Luke A. K. Blackbourn, R. Neil Dalton, David Dunger, Samira Bell, John R. Petrie, Fiona Green, Sandra MacRury, John A. McKnight, John Chalmers, Andrew Collier, Paul M. McKeigue, Helen M. Colhoun, on behalf of the Scottish Diabetes Research Network (SDRN) Type 1 Bioresource Investigators

**Affiliations:** 1 Usher Institute of Population Health Sciences and Informatics, University of Edinburgh, Edinburgh, UK; 2 MRC Institute of Genetics and Molecular Medicine, University of Edinburgh, Western General Hospital, Crewe Road South, Edinburgh, EH4 2XU UK; 3 Evelina London Children’s Hospital, Guy’s and St Thomas’ National Health Service Foundation Trust, London, UK; 4 Department of Paediatrics, University of Cambridge, Cambridge, UK; 5 Wellcome Trust–MRC Institute of Metabolic Science, University of Cambridge, Cambridge, UK; 6 Renal Unit, Ninewells Hospital, Dundee, UK; 7 Institute of Cardiovascular and Medical Sciences, University of Glasgow, Glasgow, UK; 8 Research & Development Support Unit, Dumfries & Galloway Royal Infirmary, Dumfries, UK; 9 Department of Diabetes and Cardiovascular Science, University of Highlands and Islands, Inverness, UK; 10 Western General Hospital, NHS Lothian, Edinburgh, UK; 11 Diabetes Centre, Victoria Hospital, NHS Fife, Kirkcaldy, UK; 12 School of Health and Life Sciences, Glasgow Caledonian University, Glasgow, UK; 13 Public Health, NHS Fife, Kirkcaldy, UK

**Keywords:** Clinical science, Epidemiology, Metabolomics, Nephropathy, Proteomics

## Abstract

**Aims/hypothesis:**

We examined whether candidate biomarkers in serum or urine can improve the prediction of renal disease progression in type 1 diabetes beyond prior eGFR, comparing their performance with urinary albumin/creatinine ratio (ACR).

**Methods:**

From the population-representative Scottish Diabetes Research Network Type 1 Bioresource (SDRNT1BIO) we sampled 50% and 25% of those with starting eGFR below and above 75 ml min^−1^ [1.73 m]^−2^, respectively (*N* = 1629), and with median 5.1 years of follow-up. Multiplexed ELISAs and single molecule array technology were used to measure nine serum biomarkers and 13 urine biomarkers based on our and others’ prior work using large discovery and candidate studies. Associations with final eGFR and with progression to <30 ml min^−1^ [1.73] m^−2^, both adjusted for baseline eGFR, were tested using linear and logistic regression models. Parsimonious biomarker panels were identified using a penalised Bayesian approach, and their performance was evaluated through tenfold cross-validation and compared with using urinary ACR and other clinical record data.

**Results:**

Seven serum and seven urine biomarkers were strongly associated with either final eGFR or progression to <30 ml min^−1^ [1.73 m]^−2^, adjusting for baseline eGFR and other covariates (all at *p*<2.3 × 10^−3^). Of these, associations of four serum biomarkers were independent of ACR for both outcomes. The strongest associations with both final eGFR and progression to <30 ml min^−1^ [1.73 m]^−2^ were for serum TNF receptor 1, kidney injury molecule 1, CD27 antigen, α-1-microglobulin and syndecan-1. These serum associations were also significant in normoalbuminuric participants for both outcomes. On top of baseline covariates, the *r*^2^ for prediction of final eGFR increased from 0.702 to 0.743 for serum biomarkers, and from 0.702 to 0.721 for ACR alone. The area under the receiver operating characteristic curve for progression to <30 ml min^−1^ [1.73 m]^−2^ increased from 0.876 to 0.953 for serum biomarkers, and to 0.911 for ACR alone. Other urinary biomarkers did not outperform ACR.

**Conclusions/interpretation:**

A parsimonious panel of serum biomarkers easily measurable along with serum creatinine may outperform ACR for predicting renal disease progression in type 1 diabetes, potentially obviating the need for urine testing.

**Electronic supplementary material:**

The online version of this article (10.1007/s00125-019-05081-8) contains peer-reviewed but unedited supplementary material, which is available to authorised users.

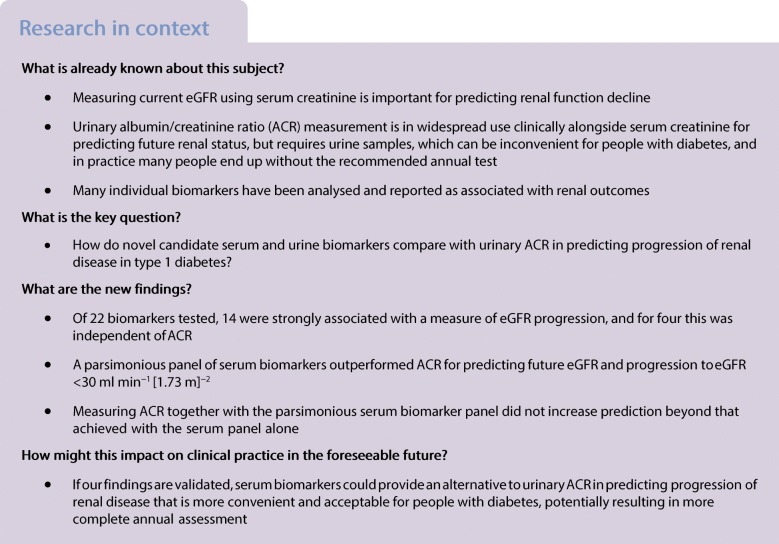



## Introduction

To facilitate the early identification of those most at risk of decline in future renal function, most clinical guidelines recommend the use of regular serum creatinine measurement and equations such as the Chronic Kidney Disease Epidemiology Collaboration (CKD-EPI) or Modification of Diet in Renal Disease (MDRD) equations for estimation of GFR [1]. The best predictor of future end-stage renal disease (ESRD) is the current eGFR and past eGFR trajectory [2]. As we recently reviewed [3], while serum cystatin C-based eGFR equations have also been proposed, at present creatinine-based equations remain the most widely used for estimating GFR [4–8]. Guidelines also advocate regular urinary testing for albumin/creatinine ratio (ACR) since there is extensive evidence that albuminuria is a strong risk factor for progression of diabetic kidney disease (DKD). However, it has been widely discussed that ACR lacks specificity and sensitivity for progressive decline in eGFR. For example, a poor positive predictive value was reported, with only about a third of those with microalbuminuria having progressive renal function decline [9]. Albumin excretion also had low sensitivity in that only about half of those with progressive renal function decline were albuminuric [9]. A further practical limitation of ACR is that obtaining urine samples for ACR adds to clinic workload and can be inconvenient and difficult for people with diabetes, resulting in suboptimal uptake. In Scotland, for example, while 86% of those with type 1 diabetes had eGFR in the past year, only 62% had ACR measured [10].

An important question is therefore whether any other prognostic biomarkers can improve on ACR or even replace it for predicting renal function decline. In a recent paper [11] we reported a large-scale discovery experiment where we assessed 297 biomarkers in two type 1 diabetes cohorts with starting eGFRs of 75 ml min^−1^ [1.73 m]^−2^ or less. We found many highly statistically significant biomarker associations with future eGFR, and that prediction of future eGFR using ACR and baseline eGFR could be improved with biomarkers. However, most of the improvement could be achieved by measuring just serum kidney injury molecule 1 (KIM-1) and CD27 antigen (CD27), a member of the TNF superfamily.

Following on from that study, we extended the evaluation to (1) include study participants with higher starting eGFRs; (2) measure the best biomarkers from our first study i.e. KIM-1 and CD27, but also measure new candidate biomarkers chosen from recent reports from large well-conducted studies; and (3) evaluate a more sensitive serum KIM-1 assay using single molecule array (SIMOA) technology, hypothesising that it would be detectable before microalbuminuria in those progressing to renal function decline. The focus of our analysis was to assess how well these biomarkers predict future eGFR and whether they could outperform or add to the prediction gained with ACR across a wide range of initial eGFRs.

## Methods

### Participants

The Scottish Diabetes Research Network Type 1 Bioresource (SDRNT1BIO) [12] is a prospective cohort study comprising 6127 people with a clinical diagnosis of type 1 diabetes mellitus, representing 25% of all adults with type 1 diabetes in Scotland, recruited between December 2010 and November 2013. On the day of recruitment (which we refer to as the study day or biosample date), clinical measurements and blood and urine samples were obtained in which serum creatinine and urinary ACR were measured. From electronic healthcare records we extracted routine health-related data retrospective and prospective to study day, as described [12]. For this study we selected 1629 individuals with eGFR 30 ml min^−1^ [1.73 m]^−2^ or above at study biosample date and with at least three prospective eGFR determinations over a period of at least 2 years or incident ESRD. These were a random sample of 50% and 25% of those with starting eGFR below and above 75 ml min^−1^ [1.73 m]^−2^, respectively.

The study was performed in accordance with the Declaration of Helsinki; all participants gave their written consent and the study protocol was approved by the local ethics and data governance committee.

### Renal outcomes and covariate data

eGFR was calculated with the CKD-EPI equation [13] using serum creatinine values directly measured and retrieved retrospectively and prospectively from medical records. These excluded readings concurrent with hospital admissions. A summary measure of the historical eGFR was obtained by computing a weighted average of all retrospective eGFR records for each person, with weights inversely related to the amount of time leading to the biosample date. Participants with no retrospective eGFR data had their historical eGFR imputed to study day eGFR. Final eGFR was defined as the median eGFR reading of the last 6 months of follow-up. Initiation of renal replacement therapy was considered to indicate an achieved eGFR of 10 ml min^−1^ [1.73 m]^−2^ and all subsequent readings were censored. The decline of renal function was estimated by fitting a simple linear regression model to the serial prospective eGFR determinations of each person. We also defined binary clinically significant progression categories of progression to <30 and <45 ml min^−1^ [1.73 m]^−2^.

ACR was measured in paired urine samples with the first taken at study day and the second several days later using the ADVIA 2400 immunoturbidimetric method for albumin and the ADVIA 2400 enzymatic method for creatinine (Siemens, Munich, Germany). These data were used for adjusting for ACR in the analyses here. In addition, longitudinal urinary ACR data were captured from the routine clinical laboratory data. Clinical record data close to study day were highly correlated (*r* = 0*.*73) with the direct measurement. At any time point, albuminuric status was defined based on the most recent available albuminuria measurement provided there was no contradictory record of that stage in the preceding or subsequent 90 days; i.e. someone who transited from normo- to microalbuminuria but then had another normoalbuminuria measurement within 90 days was assigned as having been normoalbuminuric across that period, such that transient changes in albuminuria readings were ignored. Participants were classified as normo-, micro- or macroalbuminuric according to their ACR falling in the intervals 0–3.39, 3.39–33.9 or above 33.9 mg/mmol, based on two out of three consecutive measurements before baseline.

Retrospective and prospective covariate data including drug exposures were obtained from the electronic healthcare record SCI-Diabetes as described previously [12].

### Biomarkers measured and analysed

Serum and urine biomarkers were measured on samples stored at −80°C with no prior freeze/thaw cycles. The assay methods used and the quality control performance are summarised in electronic supplementary material (ESM) Table 1. The candidate biomarkers were chosen either because we had already shown these proteins to be predictive of eGFR decline at chronic kidney disease (CKD) stage 3 or worse (serum KIM-1, CD27, α-1-microglobulin, thrombomodulin) or because they were reported from other well-conducted studies as predictive of renal disease progression (EGF and its ratio to monocyte chemotactic protein 1 [MCP-1], EGF receptor, cystatin C, macrophage inhibitory protein, matrix metalloproteinase 8, TNF receptor 1 [TNFR1]) or as strong candidates from known biology of DKD (syndecan-1), or because they are routinely measured on the same multiplex panel as a candidate (the remainder).

We used a combination of assays using (1) the Luminex platform (Austin, TX, USA) at the Clinical Laboratory Improvement Amendments (CLIA)-certified Myriad RBM laboratory (Austin, TX, USA); (2) a high-sensitivity SIMOA assay for KIM-1 at Myriad RBM that we had found detected KIM-1 in samples KIM-1 negative on their standard Luminex assay; (3) R&D Systems (Minneapolis, MN, USA) Luminex assay at the Immunoassay Biomarker Core Laboratory, University of Dundee (Dundee, UK).

Intraclass correlations were computed over 48 blinded duplicate aliquots to evaluate the reproducibility of the measurements obtained. Biomarkers were excluded from the analysis if their intraclass correlation was less than 0.4, or if over 99% of the readings in the dataset were identical due to falling below the detection threshold. Accordingly, nine serum and 13 urinary biomarkers were included in the final analyses.

Values below the detection limit were imputed to half the detection threshold. All urine biomarkers were normalised to urinary creatinine.

### Univariate analysis

To test for associations with renal outcomes, biomarkers were evaluated independently in linear models for final eGFR and logistic regression models for progression adjusted for age, sex, diabetes duration, study day eGFR and length of follow-up (basic covariates). To allow comparison with ACR, we reran the basic and biomarker models including ACR at biosample date. We further adjusted models for BMI, systolic BP, diastolic BP, HbA_1c_, HDL-cholesterol, total cholesterol, smoking status and a weighted summary of prior eGFR from retrospective records (full covariates). Prior to fitting models, all continuous covariates and biomarkers were Gaussianised and standardised to zero mean and unit standard deviation. Associations were declared significant at Bonferroni-corrected *p* = 0.05/22 = 2.3 × 10^−3^.

### Construction of parsimonious panels of biomarkers

Urine and serum biomarker sets were modelled independently from each other. As previously described [11], we adopted a Bayesian modelling approach based on hierarchical shrinkage priors, in which the clinical covariates used to control for confounding in the models were assigned a weakly informative Gaussian prior (which induces some regularisation as in ridge regression), while biomarkers were penalised through the regularised horseshoe prior (which heavily shrinks regression coefficients toward zero unless they are informative) to promote sparsity [14]. A similar approach was also adopted elsewhere for biomarker selection in the context of type 2 diabetes mellitus [15]. The hierarchical shrinkage approach was implemented using the Stan Bayesian inference framework [16], which uses Markov chain Monte Carlo (MCMC) to sample the posterior distribution of the parameters given the data and the model. The specific models implemented are available in the R package hsstan (version 0.6: https://CRAN.R-project.org/package=hsstan).

We evaluated the predictive performance of models on withdrawn data using tenfold cross-validation. For each set of baseline covariates used, we reported the difference in log-likelihood (computed on the observations withdrawn for testing from tenfold cross-validation) between the model with baseline covariates and biomarkers and the model including only the clinical covariates. For models of achieved eGFR we computed the *r*^2^ as the squared Pearson correlation coefficient between observed and predicted outcome on the test folds. For models of progression, we reported the area under the receiver operating characteristic curve (AUC) and the expected information for discrimination, Λ, expressed in bits [17]. This measure quantifies the gain in information that a set of biomarkers provides over and beyond the baseline clinical covariates. The expected information for discrimination was computed with the R package wevid (version 0.6.2: https://CRAN.R- project.org/package=wevid).

To recover a parsimonious model, we then applied projection predictive variable selection [18]. This approach is based on projecting the high-dimensional draws from the posterior of the model containing all biomarkers (full model) onto lower-dimensional subspaces corresponding to sparse candidate submodels [19, 20]. Predictions made by each candidate submodel are compared with those obtained by the full model: their discrepancy is evaluated using the Kullback–Leibler divergence, which measures the information lost when a smaller submodel is used to approximate a more complex one. By recursively choosing in a forward-selection fashion the submodel that minimises the Kullback–Leibler divergence from the full model, one can construct a series of candidate models of growing complexity. We evaluated each candidate model in terms of its relative contribution towards the performance of the full model, and plotted the relative explanatory power obtained by biomarker panels of different sizes.

## Results

### Participant characteristics

Table 1 reports the summary characteristics of those included stratified by CKD stage. The median (interquartile range [IQR]) of follow-up was 5.1 (4.4, 5.7) years. For the evaluation of progression status there were a median (IQR) of 9 (6, 14) measurements of eGFR prospectively. The median change in eGFR was a fall of 0.8 ml min^−1^ [1.73 m]^−2^ per year. Overall, 41 and 74 participants, respectively, progressed to <30 and <45 ml min^−1^ [1.73 m]^−2^ (corresponding to 2.5% and 4.7% of participants not already below these thresholds). Among the 1404 normoalbuminuric individuals at baseline, 23.1% developed albuminuria during follow-up.

**Table 1:** Participant characteristics at study day stratified by CKD stage

Covariate	Missing	G1 (*n* = 835)	G2 (*n* = 601)	G3 (*n* = 191)	All (*N* = 1629)
Main characteristics					
Age (years)	–	41.4 (30.4, 49.8)	54.8 (46.0, 63.5)	63.6 (55.3, 71.1)	48.3 (37.9, 59.2)
Sex (female), %	–	41.9	55.2	57.6	48.6
Diabetes duration (years)	–	19.0 (10.2, 28.3)	25.4 (16.5, 36.1)	32.1 (21.1, 42.4)	22.4 (12.7, 33.1)
Observability					
Retrospective study length (years)	–	5.4 (4.5, 6.0)	5.5 (5.0, 6.2)	5.5 (5.1, 6.0)	5.4 (4.8, 6.1)
Length of follow-up (years)	–	5.0 (4.3, 5.6)	5.2 (4.6, 5.7)	5.3 (4.5, 5.9)	5.1 (4.4, 5.7)
Retrospective creatinine readings (n)	–	9 (6, 14)	12 (9, 18)	16 (10, 24)	11 (7, 17)
Prospective creatinine readings (n)	–	8 (5, 11)	11 (7, 15)	15 (9, 24)	9 (6, 14)
Kidney function					
ACR (mg/mmol)	21	0.4 (0.2, 0.8)	0.4 (0.3, 1.4)	0.9 (0.4, 4.7)	0.4 (0.2, 1.1)
ACR category (normo/micro/macro),	21	91.9/7.1/1.1	85.9/10.2/3.9	71.7/15.8/12.5	87.3/9.2/3.5
Prevalent micro- or macroalbuminuria, %	–	8.1	14.1	28.8	12.8
Incident micro- or macroalbuminuria, %	–	16.9	21.1	28.8	19.9
eGFR (ml min^−1^ [1.73 m]^−2^)	–	104.4 (96.5, 114.6)	74.1 (68.0, 81.8)	51.1 (43.1, 56.0)	90.7 (70.1, 104.9)
Weighted historical eGFR (ml min^−1^ [1.73 m]^−2^)	115	107.6 (99.1, 116.6)	83.2 (76.6, 91.2)	61.3 (54.5, 70.6)	93.1 (79.2, 107.9)
Prospective eGFR slope (ml min^−1^ [1.73 m]^−2^ year^−1^)	–	−0.7 (−2.1, 0.7)	−0.7 (−2.7, 0.7)	−1.3 (−3.5, 0.7)	−0.8 (−2.5, 0.7)
Other covariates					
HbA_1c_ (mmol/mol)	3	70 (62, 81)	68 (60, 78)	68 (60, 80)	69 (61, 80)
HbA_1c_ (%)	3	8.6 (7.8, 9.6)	8.4 (7.6, 9.3)	8.4 (7.6, 9.5)	8.5 (7.7, 9.5)
HDL-cholesterol (mmol/l)	54	1.5 (1.2, 1.8)	1.6 (1.3, 1.9)	1.5 (1.2, 1.9)	1.5 (1.2, 1.8)
LDL-cholesterol (mmol/l)	863	2.5 (2.0, 3.0)	2.3 (1.9, 2.9)	2.0 (1.6, 2.4)	2.4 (1.9, 2.9)
Total cholesterol (mmol/l)	21	4.6 (4.0, 5.2)	4.6 (4.0, 5.1)	4.2 (3.7, 4.9)	4.5 (4.0, 5.2)
BMI (kg/m^2^)	9	26.0 (23.3, 29.5)	26.9 (24.5, 30.1)	27.1 (24.0, 31.0)	26.6 (23.8, 29.9)
Diastolic BP (mmHg)	6	75 (68, 81)	76 (68, 81)	70 (63, 80)	75 (68, 81)
Systolic BP (mmHg)	6	128 (119, 137)	133 (123, 146)	137 (124, 148)	130 (120, 141)
Ever smoker, %	–	61.9	65.2	72.3	64.3
On any anti-hypertensive treatment, %	–	28.1	58.7	85.9	46.3
On ACEi or ARB, %	–	26.1	52.9	77.5	42.1

We report frequency (as %) for categorical variables and median (IQR) for continuous variables

CKD stages are defined according to ranges of eGFR in ml min^−1^ [1.73 m]^−2^: G1: >90; G2: 60–90; G3: 30–60

Participants at stage G4 (eGFR = 15–30; *n* = 2) are not reported as a separate column

ACEi, ACE inhibitor; ARB, angiotensin receptor blocker

### Biomarkers explored

ESM Table 1 shows the median, IQR and range of the biomarkers analysed in this study. All candidate biomarkers showed good intraclass correlation coefficients (≥0*.*75) when measured on blinded duplicate samples. ESM Table 2 lists the biomarkers that were excluded from the analysis.

**Fig. 1:**
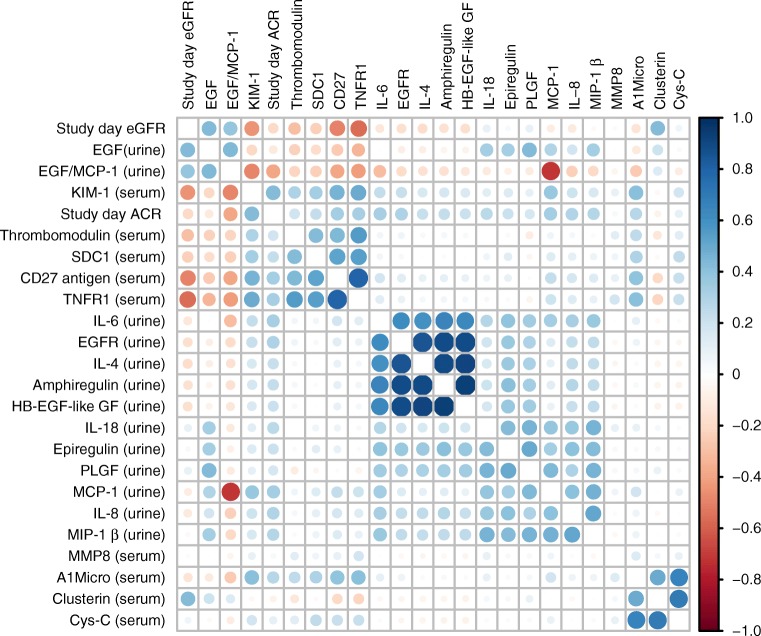
Correlation matrix ordered by hierarchical clustering. A1Micro, α-1-microglobulin; Cys-C, cystatin C; GF, growth factor; HB, heparin-binding; MMP8, matrix metalloproteinase-8; PLGF, placenta growth factor; SDC1, syndecan 1

The correlation matrix for the biomarkers ordered by hierarchical clustering is shown in Fig. 1. Four distinct clusters are seen; for example, we see that cystatin C is moderately correlated with α-1-microglobulin and clusterin, but only weakly correlated with other biomarkers. TNFR1 and CD27 were very strongly correlated with each other and moderately correlated with KIM-1.

### Univariate associations with final eGFR

Seven serum and six urine biomarkers, including the ratio of urinary EGF to MCP-1, were strongly associated with final eGFR in models adjusted for basic covariates including baseline eGFR (all at *p*<2.3 × 10^−3^; Table 2). Of these, nine remained significantly associated with final eGFR after further adjustment for ACR at biosample date. The most strongly associated biomarkers, TNFR1, KIM-1, CD27, syndecan-1, α-1-microglobulin and urinary EGF/MCP-1 ratio, were also significantly associated with final achieved eGFR when restricted to those normoalbuminuric at biosample date and even when adjusted for ACR in this normoalbuminuric range (data not shown). Furthermore, other than α-1-microglobulin, these biomarkers were also highly significantly associated with final eGFR at follow-up adjusted for ACR at baseline when restricted to those with eGFR ≥90 ml min^−1^ [1.73 m]^−2^ at baseline (data not shown). We confirmed that the associations were similar when adjusted for use of ACE inhibitors or angiotensin receptor blockers.

**Table 2:** Associations of each biomarker (considered separately) with final eGFR

Biomarker	Basic covariates		Basic covariates + ACR		Full covariates	
	β (95% CI)	*p* value	β (95% CI)	*p* value	β (95% CI)	*p* value
Serum biomarkers						
TNFR1	−0.20 (−0.24, −0.17)	1*.*4 *×* 10^*−*35^	−0.17 (−0.20, −0.14)	3.0 *×* 10^*−*24^	−0.12 (−0.16, −0.09)	3.2 × 10^*−*14^
KIM-1	−0.18 (−0.21, −0.16)	4*.*8 *×* 10^*−*35^	−0.14 (−0.17, −0.11)	1*.*7 *×* 10^*−*19^	−0.14 (−0.17, −0.11)	7.0 *×* 10^*−*20^
CD27	−0.17 (−0.20, −0.14)	7.1 *×* 10^*−*29^	−0.14 (−0.17, −0.11)	1*.*8 *×* 10^*−*18^	−0.11 (−0.13, −0.08)	1*.*4 *×* 10^*−*12^
α-1-microglobulin	−0.12 (−0.14, −0.09)	1*.*1 *×* 10^*−*17^	−0.08 (−0.11, −0.06)	7.4 *×* 10^*−*10^	−0.07 (−0.10, −0.05)	2*.*9 *×* 10^*−*08^
Syndecan 1	−0.12 (−0.15, −0.09)	5*.*1 *×* 10^*−*17^	−0.09 (−0.12, −0.06)	1.0 *×* 10^*−*10^	−0.07 (−0.10, −0.04)	1.0 *×* 10^*−*07^
Thrombomodulin	−0.10 (−0.13, −0.07)	2*.*0 *×* 10^*−*11^	−0.07 (−0.10, −0.05)	2*.*6 *×* 10^*−*07^	−0.05 (−0.07, −0.02)	3*.*1 *×* 10^*−*04^
Cystatin C	−0.05 (−0.07, −0.02)	5*.*9 *×* 10^*−*04^	−0.03 (−0.06, 0.00)	2*.*8 *×* 10^*−*02^	−0.03 (−0.06, −0.01)	1*.*3 *×* 10^*−*02^
Matrix metalloproteinase-8	−0.04 (−0.07, −0.01)	2*.*6 *×* 10^*−*03^	−0.03 (−0.06, −0.01)	1*.*0 *×* 10^*−*02^	−0.02 (−0.04, 0.00)	1*.*1 *×* 10^*−*01^
Clusterin	0.01 (−0.02, 0.04)	4*.*5 *×* 10^*−*01^	0.02 (−0.01, 0.04)	2*.*9 *×* 10^*−*01^	0.00 (−0.02, 0.03)	7*.*9 *×* 10^*−*01^
Urine biomarkers						
EGF/MCP-1 ratio	0.16 (0.13, 0.19)	1*.*7 *×* 10^*−*27^	0.12 (0.09, 0.15)	6.1 *×* 10^*−*15^	0.10 (0.07, 0.13)	1.1 *×* 10^*−*12^
MCP-1	−0.10 (−0.13, −0.08)	2*.*2 *×* 10^*−*14^	−0.06 (−0.09, −0.04)	4.6 *×* 10^*−*06^	−0.07 (−0.09, −0.04)	4.8 *×* 10^*−*07^
IL-8	−0.07 (−0.10, −0.05)	6*.*5 *×* 10^*−*07^	−0.03 (−0.06, 0.00)	5.3 *×* 10^*−*02^	−0.02 (−0.05, 0.01)	1*.*5 *×* 10^*−*01^
EGF	0.07 (0.04, 0.10)	1*.*0 *×* 10^*−*06^	0.07 (0.04, 0.10)	6.3 *×* 10^*−*06^	0.05 (0.02, 0.07)	1*.*1 *×* 10^*−*03^
EGF receptor	−0.05 (−0.08, −0.03)	1.0 *×* 10^*−*04^	−0.01 (−0.04, 0.01)	3.6 *×* 10^*−*01^	−0.01 (−0.03, 0.02)	6.1 *×* 10^*−*01^
IL-18	−0.05 (−0.08, −0.02)	5.0 *×* 10^*−*04^	−0.01 (−0.03, 0.02)	7*.*0 *×* 10^*−*01^	0.00 (−0.03, 0.02)	7.1 *×* 10^*−*01^
IL-6	−0.04 (−0.07, −0.01)	2*.*6 *×* 10^*−*03^	0.00 (−0.02, 0.03)	7.8 *×* 10^*−*01^	0.00 (−0.02, 0.03)	7*.*2 *×* 10^*−*01^
Macrophage inflammatory protein-1 β	−0.04 (−0.07, −0.01)	5*.*2 *×* 10^*−*03^	0.00 (−0.02, 0.03)	8.4 *×* 10^*−*01^	0.00 (−0.02, 0.03)	8.4 *×* 10^*−*01^
Amphiregulin	−0.04 (−0.06, −0.01)	1.0 *×* 10^*−*02^	0.00 (−0.03, 0.02)	7.5 *×* 10^*−*01^	0.01 (−0.02, 0.03)	6.6 *×* 10^*−*01^
Placenta growth factor	−0.03 (−0.05, 0.00)	5.3 *×* 10^*−*02^	0.00 (−0.02, 0.03)	8.2 *×* 10^*−*01^	0.00 (−0.02, 0.03)	9*.*5 *×* 10^*−*01^
IL-4	−0.02 (−0.05, 0.00)	8.2 *×* 10^*−*02^	0.01 (−0.02, 0.03)	6.9 *×* 10^*−*01^	0.02 (−0.01, 0.04)	2*.*3 *×* 10^*−*01^
Epiregulin	−0.02 (−0.05, 0.00)	8*.*6 *×* 10^*−*02^	0.01 (−0.02, 0.04)	4.7 *×* 10^*−*01^	0.01 (−0.02, 0.03)	4.8 *×* 10^*−*01^
Heparin-binding EGF-like growth factor	−0.02 (−0.05, 0.01)	1*.*2 *×* 10^*−*01^	0.01 (−0.02, 0.03)	6.5 *×* 10^*−*01^	0.02 (−0.01, 0.04)	2*.*2 *×* 10^*−*01^

Regression coefficients are per unit SD of Gaussianised biomarker

Basic clinical covariates: age, sex, diabetes duration, study day eGFR, length of follow-up

Full clinical covariates: age, sex, diabetes duration, study day eGFR, length of follow-up, ACR, BMI, diastolic BP, systolic BP, HbA_1c_, HDL-cholesterol, total cholesterol, smoking status, weighted average of historical eGFR

Using progression status to <30 or <45 ml min^−1^ [1.73 m]^−2^ as the outcome, a very similar pattern was seen for serum biomarkers (ESM Tables 3 and 4). For example, adjusted for basic covariates and ACR, the odds of progression to <30 ml min^−1^ [1.73 m]^−2^ were 5.80-fold for every 1 SD in Gaussianised TNFR1, and 2.05-fold per SD of KIM-1 (ESM Table 3). Among the urinary biomarkers, EGF/MCP-1 ratio was associated with progression, but when adjusted for ACR the association remained significant for progression to <45 but not <30 ml min^−1^ [1.73 m]^−2^. When restricted to those with normo- or microalbuminuria at baseline, a very similar pattern of association with progression was seen. Among those with macroalbuminuria at baseline, significant associations with outcomes were not found, but the sample size was very small for testing this.

### Panels of biomarkers for predicting eGFR

Table 3 summarises the cross-validated performance of the linear regression models for prediction of final eGFR using all of the serum or urine biomarkers (in models with hierarchical shrinkage priors) on top of clinical covariates, with and without further inclusion of ACR at biosample date. Starting from basic covariates, the *r*^2^ for prediction of final eGFR increased from 0.702 to 0.743 for serum and to 0.721 for urine biomarkers, compared with an increase to 0.722 for ACR alone. Thus, the serum biomarkers alone outperform ACR alone. As shown in Table 3, the model including serum biomarkers with ACR is better than ACR alone.

**Table 3:** Cross-validated performance of models for prediction of final eGFR

Model	Basic covariates		Basic covariates + ACR		Full covariates	
	ΔLoglik	*r*^2^ (95% PI)	ΔLoglik	*r*^2^ (95% PI)	ΔLoglik	*r*^2^ (95% PI)
Clinical covariates only	–	0.702 (0.700, 0.704)	–	0.722 (0.720, 0.724)	–	0.758 (0.756, 0.761)
Serum biomarkers	120.9	0.743 (0.740, 0.746)	73.3	0.746 (0.743, 0.749)	56.3	0.775 (0.772, 0.777)
Urine biomarkers	54.2	0.721 (0.718, 0.724)	21.6	0.729 (0.726, 0.732)	19.2	0.764 (0.761, 0.767)

Basic and full clinical covariates are listed in the footnotes to Table 2

ΔLoglik, difference in test log-likelihood (natural logarithm) with respect to the model containing only clinical covariates; PI, posterior uncertainty interval

Similarly, from Table 4 the AUC for progression to <30 ml min^−1^ [1.73 m]^−2^ was 0.911 using ACR with basic covariates, but was 0.953 with serum biomarkers and basic covariates, and did not increase further after adding ACR to serum biomarkers. Using the expected information for discrimination, Λ, Table 4 shows that serum biomarkers contain almost one extra bit of information for the prediction of progression to <30 ml min^−1^ [1.73 m]^−2^ than does ACR (4.06 vs 3.23 bits).

**Table 4:** Cross-validated performance of models for prediction of final eGFR being <30 or <45 ml min^−1^ [1.73 m]^−2^, overall and stratified by albuminuric status at study day

Model	Basic covariates			Basic covariates + ACR			Full covariates		
	ΔLoglik	AUC (95% PI)	Λ	ΔLoglik	AUC (95% PI)	Λ	ΔLoglik	AUC (95% PI)	Λ
Final eGFR <30 (*n* = 1627, 41 events)									
Clinical covariates only	–	0.876 (0.858, 0.890)	2.18	–	0.911 (0.895, 0.924)	3.23	–	0.929 (0.912, 0.943)	3.93
Serum biomarkers	35.3	0.953 (0.940, 0.965)	4.06	11.2	0.952 (0.939, 0.965)	4.08	0.8	0.940 (0.920, 0.956)	4.28
Urine biomarkers	3.2	0.879 (0.852, 0.901)	2.52	−13.8	0.892 (0.866, 0.913)	2.84	−0.2	0.929 (0.912, 0.943)	3.92
Final eGFR <30 in normo-/microalbuminuric (*n* = 1571, 18 events)									
Clinical covariates only	–	0.788 (0.737, 0.836)	1.32	–	0.793 (0.740, 0.845)	1.43	–	0.818 (0.757, 0.873)	2.10
Serum biomarkers	9.3	0.861 (0.807, 0.909)	2.20	7.3	0.856 (0.799, 0.908)	2.14	−2.6	0.815 (0.760, 0.871)	2.20
Urine biomarkers	−0.3	0.786 (0.732, 0.840)	1.31	−1.2	0.787 (0.732, 0.840)	1.32	0.3	0.819 (0.761, 0.873)	2.10
Final eGFR <30 in macroalbuminuric (*n* = 56, 23 events)									
Clinical covariates only	–	0.750 (0.692, 0.809)	1.33	–	0.801 (0.743, 0.852)	1.95	–	0.771 (0.697, 0.843)	2.56
Serum biomarkers	4.3	0.830 (0.755, 0.896)	2.31	1.6	0.835 (0.760, 0.895)	2.33	−8.6	0.764 (0.685, 0.839)	2.46
Urine biomarkers	−1.0	0.770 (0.697, 0.835)	1.45	−4.7	0.765 (0.686, 0.833)	1.41	−7.6	0.758 (0.677, 0.834)	2.24
Final eGFR <45 (*n* = 1573, 74 events)									
Clinical covariates only	–	0.840 (0.830, 0.849)	1.54	–	0.889 (0.881, 0.897)	2.31	–	0.901 (0.891, 0.911)	2.68
Serum biomarkers	33.6	0.899 (0.887, 0.911)	2.49	8.3	0.907 (0.894, 0.918)	2.59	5.4	0.914 (0.901, 0.926)	2.96
Urine biomarkers	15.3	0.879 (0.866, 0.890)	1.98	−5.5	0.890 (0.877, 0.902)	2.22	−0.3	0.901 (0.891, 0.912)	2.68
Final eGFR <45 in normo-/microalbuminuric (*n* = 1529, 53 events)									
Clinical covariates only	–	0.857 (0.843, 0.870)	1.70	–	0.866 (0.854, 0.878)	1.93	–	0.876 (0.858, 0.891)	2.33
Serum biomarkers	16.3	0.891 (0.877, 0.904)	2.31	9.9	0.890 (0.875, 0.904)	2.30	5.0	0.891 (0.872, 0.908)	2.63
Urine biomarkers	−2.4	0.852 (0.834, 0.869)	1.71	−9.7	0.849 (0.831, 0.867)	1.75	−1.6	0.872 (0.851, 0.890)	2.34
Final eGFR <45 in macroalbuminuric (*n* = 44, 21 events)									
Clinical covariates only	–	0.538 (0.429, 0.640)	0.17	–	0.529 (0.416, 0.634)	0.19	–	0.561 (0.472, 0.644)	0.25
Serum biomarkers	−10.1	0.510 (0.400, 0.627)	0.07	−9.3	0.512 (0.395, 0.634)	0.07	−30.1	0.536 (0.437, 0.634)	0.22
Urine biomarkers	−2.7	0.541 (0.422, 0.646)	0.18	−0.1	0.540 (0.428, 0.658)	0.17	−94.1	0.479 (0.379, 0.578)	−0.35

Basic and full clinical covariates are listed in the footnotes to Table 2

ΔLoglik, difference in test log-likelihood (natural logarithm) with respect to the model containing only clinical covariates; PI, posterior uncertainty interval; Λ, expected information for discrimination in bits

The same conclusion is reached using progression to <45 ml min^−1^ [1.73 m]^−2^, and also when restricted to those with normo- or microalbuminuria at baseline. Among those with macroalbuminuria, however, there was no evidence of serum biomarkers outperforming or improving prediction beyond ACR. There was no evidence of urinary biomarkers outperforming ACR for progression in any stratum.

**Fig. 2:**
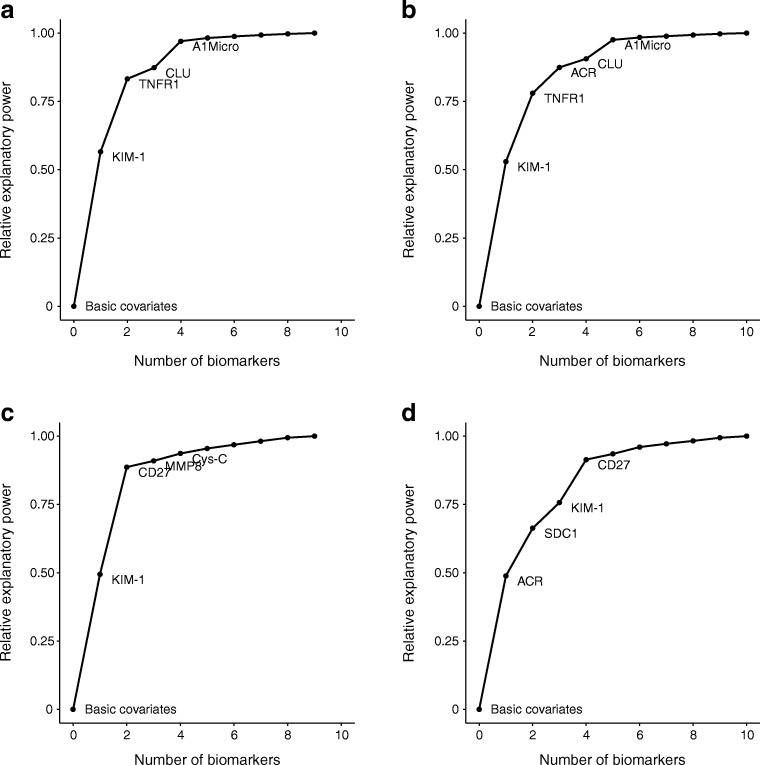
Contribution of biomarker sets to prediction of final eGFR (**a**, **b**) or final eGFR <30 (**c**, **d**) when starting from a model containing basic covariates (age, sex, diabetes duration, study day eGFR and length of follow-up). Variable selection was based on: serum biomarkers (**a**, **c**); and serum biomarkers and ACR (**b**, **d**). A1Micro, α-1-microglobulin; CLU, clusterin; Cys-C, cystatin C; MMP8, matrix metalloproteinase-8; SDC1, syndecan 1

By applying the projection predictive variable selection approach, we determined a ranking of the biomarkers within each platform. Figure 2 displays the ranking of the first few serum biomarkers when predicting final eGFR or progression to <30 ml min^−1^ [1.73 m]^−2^, starting from the basic set of covariates. We also treated baseline ACR as another biomarker (Fig. 2b,d). For final eGFR, TNFR1 and KIM-1 together account for more than 75% of the information contained in the whole set of serum biomarkers independently of ACR, with clusterin and α-1-microglobulin making a small contribution beyond this (Fig. 2a,b). For progression to <30 ml min^−1^ [1.73 m]^−2^, KIM-1 and CD27 (which is very strongly correlated with TNFR1) contain most of the predictive information (Fig. 2c). KIM-1, CD27 and syndecan 1 also contribute to the total prediction on top of ACR (Fig. 2d).

## Discussion

The key findings from this study across the range of CKD stages were that serum biomarkers improve the prediction of future eGFR and progression to <30 ml min^−1^ [1.73 m]^−2^ beyond baseline eGFR. As in our past studies [11, 21, 22], a large number of the biomarkers evaluated showed highly significant associations with eGFR and its decline. However, almost all of the predictive information could be obtained using just a few of these intercorrelated biomarkers. Here, we found KIM-1 measured by SIMOA assay and either CD27 or TNFR1 to contain most of the predictive information. CD27 and TNFR1 are very highly correlated (*r* = 0*.*80) and are both members of the TNF superfamily and therefore likely interchangeable as predictive biomarkers. Beyond these, there is some predictive information in syndecan-1, clusterin or α-1-microglobulin.

An important emphasis of the study reported here was a comparison of biomarkers with the performance of the widely accepted measurement ACR. In this regard, we found that a parsimonious panel of serum biomarkers performed better than ACR for predicting eGFR and eGFR progression to <30 ml min^−1^ [1.73 m]^−2^. Indeed, serum biomarkers contained almost double the prediction information (measured in bits) than ACR for progression to <30 ml min^−1^ [1.73 m]^−2^. The serum biomarkers were predictive of final eGFR in those with starting eGFR above 90 ml min^−1^ [1.73 m]^−2^, suggesting that they rise before ACR does and may be more sensitive to early renal damage. Of note, the urinary biomarkers did not consistently outperform ACR.

These data challenge the place of ACR in our clinical management of people with diabetes. Most clinical guidelines now recommend annual measurement of ACR in people with diabetes. Our data suggest that measuring just KIM-1 and TNFR1 or CD27 in the same sample might obviate the need for doing a urine sample collection and then measuring ACR. Collecting urine samples is time intensive in outpatient clinics and many people miss out on annual screening because they do not bring or cannot produce a urine sample. As we note above, while in Scotland we achieve high rates of annual eGFR measurement, ACR capture rates are much lower [10]. Additional differences in reporting rates for ACR than eGFR exist elsewhere, e.g. in the National Diabetes Audit in England and Wales and in reports from Denmark [23, 24]. Clearly, further validation is required before advocating replacing ACR. Longitudinal analyses of repeat measurements of these biomarkers, for example, are needed to establish their variability and thresholds for specific risk levels warranting clinical action (akin to the microalbuminuria thresholds).

Replication in different cohorts across the range of eGFR, using a variety of assay methods, and testing for prediction of ESRD are also needed. Moreover, at the present time these biomarkers are much more expensive to assay than ACR and are not routinely available on many commonly used clinically certified assay platforms. Some may argue that ACR is also measured because it is a biomarker of more widespread vascular disease and mortality independently of renal function: this should also be shown for candidate replacement serum biomarkers. Nonetheless, at the very least our data suggest that further studies to evaluate replacing urinary ACR with serum biomarkers are warranted, given the logistic and predictive advantages they may offer.

Serum biomarkers might be useful on top of ACR rather than instead of it. In regard to the latter, it is important to note that these biomarkers also added to the prediction of final eGFR or progression even when ACR was included in the model. Furthermore, they also predicted progression to <30 ml min^−1^ [1.73 m]^−2^ in those normoalbuminuric or microalbuminuric at baseline, and did so independently of ACR. This suggests they may be useful even when ACR has already been measured and found not to be in the macroalbuminuric range. Once macroalbuminuria has been detected, however, they do not improve prediction further. Such people with macroalbuminuria would in any case be managed uniformly as a high-risk group, and it is questionable whether any further risk discrimination among them would alter clinical practice.

Our study has used eGFR and its progression to <30 and <45 ml min^−1^ [1.73 m]^−2^ as endpoints. Of course, as we recently reviewed, creatinine-based eGFR is itself an imperfect measure of underlying GFR, especially at early stages of renal function decline [3]. Ideally, biomarker studies would also evaluate prediction of harder endpoints such as ESRD. However, to evaluate prediction from early stages of renal function decline to ESRD requires large unselected cohorts of people with type 1 diabetes to be followed for many years for sufficient ESRD endpoints to accrue. Such studies are logistically challenging and usually have very few ESRD endpoints in those with eGFR >60 ml min^−1^ [1.73 m]^−2^ at baseline [25, 26]. Furthermore, it requires that we generalise from samples taken several decades ago to the contemporary state of diabetes. Therefore, studies such as ours with intermediate endpoints are also needed. It should be noted that the imprecision with which eGFR quantifies underlying GFR means that power to detect biomarker associations is reduced rather than false positive associations being detected. Our study will accrue ESRD endpoints as we continue follow-up, eventually allowing associations with this endpoint to be confirmed. Further analyses will also attempt to disentangle the contribution of prediction of intervening acute renal failure events to overall prediction of decline as events accrue. Another limitation of our study is that we were not able to assess incremental prediction on top of the validated kidney failure risk equation [27], as calcium and phosphate data were not available in most participants.

A key strength of our study was the deliberate use of samples from a wide range of starting eGFR. Another key strength of our study is the use of advanced statistical methods that avoid over-optimistic assessments of prediction. These include cross-validation and use of penalised models that account for the high number of analytes being evaluated and can better handle correlations between predictors.

### Conclusions

In summary, a parsimonious panel of serum biomarkers measurable along with creatinine may outperform ACR for predicting renal disease progression from early CKD stages in type 1 diabetes, and with further development might obviate the need for urine testing.

## Electronic supplementary material

ESM Tables (PDF 252 kb)

## Data Availability

We do not have governance permissions to share individual level data on which these analyses were conducted since they derive from clinical record data. However, for any bona fide requests to audit the validity of the analyses, the verifiable research pipeline that we operate means that one can request to view the analyses being run and the same resulting tabulations by contacting the corresponding author. We are also happy to share summary statistics for those wishing to conduct meta-analyses with other studies.

## Abbreviations

ACR: Albumin/creatinine ratio
AUC: Area under the receiver operating characteristic curve
CD27: CD27 antigen
CKD: Chronic kidney disease
CKD-EPI: Chronic Kidney Disease Epidemiology Collaboration
DKD: Diabetic kidney disease
ESRD: End-stage renal disease
IQR: Interquartile range
KIM-1: Kidney injury molecule 1
MCP-1: Monocyte chemotactic protein 1
SIMOA: Single molecule array
TNFR1: TNF receptor 1
