# Supplementary material for: Comparison of serum and urinary biomarker panels with albumin/creatinine ratio in the prediction of renal function decline in type 1 diabetes
Source: Diabetologia. 2020 Jan 8;63(4):788–98. doi: 10.1007/s00125-019-05081-8 (PMC7054370; doi:10.1007/s00125-019-05081-8)
Supplement: Supplementary file 1 — (PDF 252 kb) [file 125_2019_5081_MOESM1_ESM.pdf]

Electronic Supplementary Materials
Comparison of serum and urinary biomarker panels with albumin/creatinin ratio in the prediction of renal function decline in type 1 diabetes

Marco Colombo, Stuart J. McGurnaghan, Luke A.K. Blackbourn, R. Neil Dalton, David Dunger, Samira Bell, John R. Petrie, Fiona Green, Sandra MacRury, John A. McKnight, John Chalmers, Andrew Collier, Paul M. McKeigue and Helen M. Colhoun on behalf of the Scottish Diabetes Research Network (SDRN) Type 1 Bioresource Investigators

ESM Table 1: Median, interquartile range (IQR), range and intraclass correlations (ICC) for the biomarkers analyzed in the study

Biomarker	Laboratory	Unit	Median (IQR)	Range	ICC
**Serum biomarkers**					
Alpha-1-Microglobulin	Myriad RBM	µg/ml	18.0 (14.0, 23.0)	4.3 – 77.5	0.82
CD27 antigen	Corelabs	pg/ml	7144.8 (5555.8, 9619.2)	2689.3 – 41257.5	0.98
Clusterin	Myriad RBM	µg/ml	251.0 (196.0, 338.0)	52.0 – 1200.0	0.65
Cystatin-C	Myriad RBM	ng/ml	906.0 (709.0, 1160.0)	333.0 – 3280.0	0.78
Kidney Injury Molecule-1	Myriad RBM	pg/ml	115.0 (68.0, 214.0)	12.0 – 5830.0	0.99
Matrix Metalloproteinase-8	Corelabs	pg/ml	739.2 (493.2, 1105.5)	65.7 – 56998.6	0.94
Syndecan 1	Corelabs	pg/ml	1865.9 (1530.4, 2305.4)	516.6 – 7541.1	0.86
Thrombomodulin	Corelabs	pg/ml	6204.7 (4963.2, 7844.4)	274.6 – 64982.8	0.97
Tumour Necrosis Factor Receptor 1	Corelabs	pg/ml	986.8 (804.4, 1288.5)	361.7 – 5633.5	0.97
**Urine biomarkers**					
Amphiregulin	Myriad RBM	pg/ml	28.0 (28.0, 28.0)	28.0 – 382.0	0.97
EGF/MCP-1 ratio	Myriad RBM	ratio	121.2 (68.6, 214.0)	0.3 – 2225.9	—
Epidermal Growth Factor	Myriad RBM	pg/ml	18600.0 (8560.0, 35900.0)	433.0 – 86000.0	0.96
Epidermal Growth Factor Receptor	Myriad RBM	pg/ml	15.5 (15.5, 15.5)	15.5 – 450.0	0.76
Epiregulin	Myriad RBM	pg/ml	6.6 (3.2, 11.0)	3.2 – 484.0	0.96
Heparin-Binding EGF-Like Growth Factor	Myriad RBM	pg/ml	43.0 (39.0, 48.0)	15.0 – 64.0	0.66
Interleukin-18	Myriad RBM	pg/ml	23.0 (6.0, 49.0)	6.0 – 1460.0	0.98
Interleukin-4	Myriad RBM	pg/ml	9.0 (9.0, 9.0)	9.0 – 11100.0	—
Interleukin-6	Myriad RBM	pg/ml	0.8 (0.8, 0.8)	0.8 – 570.0	0.95
Interleukin-8	Myriad RBM	pg/ml	6.1 (1.6, 26.0)	0.7 – 3180.0	0.99
Macrophage Inflammatory Protein-1 beta	Myriad RBM	pg/ml	33.0 (17.0, 58.5)	8.0 – 661.0	0.92
Monocyte Chemotactic Protein 1	Myriad RBM	pg/ml	166.5 (74.0, 313.2)	27.0 – 4770.0	0.92
Placenta Growth Factor	Myriad RBM	pg/ml	52.0 (33.0, 80.0)	12.0 – 1070.0	0.92

Median, IQR and ranges for urine biomarkers are shown prior to normalization for urinary creatinine
Intraclass correlations were computed over 48 blinded duplicate samples
Interleukin-4 had no variation within the duplicate samples

ESM Table 2: Biomarkers removed from the analysis and reason

Biomarker	Laboratory	Reason
**Serum biomarkers**		
uPAR	Corelabs	Intraclass correlation < 0.4
**Urine biomarkers**		
Granulocyte-Macrophage Colony-Stimulating Factor	Myriad RBM	All identical readings
Interferon gamma	Myriad RBM	All identical readings
Interleukin-10	Myriad RBM	Excessive left censoring
Interleukin-2	Myriad RBM	Excessive left censoring
Interleukin-3	Myriad RBM	Excessive left censoring
Interleukin-5	Myriad RBM	Excessive left censoring
Interleukin-7	Myriad RBM	Excessive left censoring
Macrophage Inflammatory Protein-1 alpha	Myriad RBM	Excessive left censoring
Platelet-Derived Growth Factor BB	Myriad RBM	Excessive left censoring
Tenascin-C	Myriad RBM	Excessive left censoring
Tumour Necrosis Factor alpha	Myriad RBM	All identical readings
Tumour Necrosis Factor beta	Myriad RBM	Excessive left censoring

ESM Table 3: Associations of each biomarker (considered separately) with having an achieved eGFR < 30, overall and stratified by albuminuria status at study day

	Overall (N=1627, 41 events)				Normo/micro (n=1571, 18 evs)		Macro (n=56, 23 events)	
	Basic covariates		Basic covariates + ACR		Basic covariates		Basic covariates	
Biomarker	OR (95% CI)	*p* value	OR (95% CI)	*p* value	OR (95% CI)	*p* value	OR (95% CI)	*p* value
**Serum biomarkers**								
Alpha-1-Microglobulin	2.43 (1.71, 3.47)	9.3×10^{-07}	1.46 (1.00, 2.23)	6.4×10^{-02}	2.15 (1.42, 3.12)	9.0×10^{-05}	0.97 (0.37, 2.42)	9.5×10^{-01}
CD27 antigen	15.8 (7.77, 35.60)	8.0×10^{-13}	9.43 (4.37, 22.23)	5.0×10^{-08}	8.08 (3.72, 19.29)	4.8×10^{-07}	47.3 (4.86, 2274.39)	1.1×10^{-02}
Clusterin	1.01 (0.62, 1.62)	9.7×10^{-01}	0.88 (0.50, 1.53)	6.6×10^{-01}	1.20 (0.62, 2.23)	5.8×10^{-01}	0.60 (0.23, 1.38)	2.5×10^{-01}
Cystatin-C	1.83 (1.15, 2.98)	1.3×10^{-02}	1.44 (0.85, 2.48)	1.8×10^{-01}	1.89 (1.02, 3.65)	5.0×10^{-02}	1.13 (0.39, 2.96)	8.1×10^{-01}
Kidney Injury Molecule-1	3.12 (2.23, 4.50)	1.8×10^{-10}	2.05 (1.40, 3.08)	3.5×10^{-04}	3.10 (2.02, 4.93)	4.6×10^{-07}	1.42 (0.67, 3.32)	3.7×10^{-01}
Matrix Metalloproteinase-8	1.01 (0.71, 1.44)	9.6×10^{-01}	0.93 (0.63, 1.37)	7.2×10^{-01}	0.93 (0.57, 1.50)	7.5×10^{-01}	0.81 (0.40, 1.56)	5.4×10^{-01}
Syndecan 1	4.71 (2.91, 7.98)	1.4×10^{-09}	3.32 (1.96, 5.86)	1.6×10^{-05}	3.43 (1.93, 6.40)	5.0×10^{-05}	4.79 (1.56, 20.92)	1.5×10^{-02}
Thrombomodulin	2.83 (1.74, 4.80)	5.8×10^{-05}	2.01 (1.18, 3.63)	1.5×10^{-02}	2.59 (1.38, 5.15)	4.5×10^{-03}	1.64 (0.58, 5.24)	3.8×10^{-01}
Tumour Necrosis Factor Receptor 1	12.1 (5.84, 27.50)	2.3×10^{-10}	5.80 (2.61, 14.08)	4.2×10^{-05}	4.73 (2.14, 11.20)	2.2×10^{-04}	14.7 (2.52, 206.31)	1.3×10^{-02}
**Urine biomarkers**								
Amphiregulin	1.29 (0.86, 1.94)	2.1×10^{-01}	1.00 (0.67, 1.51)	9.9×10^{-01}	0.89 (0.51, 1.54)	6.8×10^{-01}	1.36 (0.60, 3.32)	4.7×10^{-01}
EGF/MCP-1 ratio	0.30 (0.19, 0.47)	4.2×10^{-07}	0.57 (0.33, 0.96)	3.8×10^{-02}	0.74 (0.42, 1.27)	2.8×10^{-01}	0.10 (0.02, 0.36)	2.5×10^{-03}
Epidermal Growth Factor	0.71 (0.44, 1.12)	1.4×10^{-01}	0.83 (0.49, 1.37)	4.7×10^{-01}	1.06 (0.57, 1.96)	8.5×10^{-01}	0.49 (0.16, 1.29)	1.7×10^{-01}
Epidermal Growth Factor Receptor	2.17 (1.49, 3.21)	6.6×10^{-05}	1.24 (0.84, 1.89)	2.9×10^{-01}	0.91 (0.52, 1.55)	7.2×10^{-01}	2.40 (1.06, 6.32)	4.9×10^{-02}
Epiregulin	1.35 (0.92, 2.01)	1.3×10^{-01}	0.99 (0.65, 1.51)	9.6×10^{-01}	1.18 (0.70, 2.00)	5.4×10^{-01}	1.07 (0.49, 2.43)	8.6×10^{-01}
Heparin-Binding EGF-Like Growth Factor	1.18 (0.80, 1.74)	3.9×10^{-01}	1.02 (0.67, 1.53)	9.4×10^{-01}	0.85 (0.50, 1.45)	5.6×10^{-01}	1.52 (0.63, 3.94)	3.7×10^{-01}
Interleukin-18	1.86 (1.26, 2.77)	2.0×10^{-03}	1.16 (0.75, 1.80)	5.0×10^{-01}	1.18 (0.70, 2.00)	5.4×10^{-01}	1.35 (0.63, 2.86)	4.3×10^{-01}
Interleukin-4	1.41 (0.97, 2.05)	7.5×10^{-02}	1.18 (0.78, 1.79)	4.3×10^{-01}	0.95 (0.56, 1.60)	8.3×10^{-01}	2.50 (1.00, 8.14)	7.9×10^{-02}
Interleukin-6	2.19 (1.50, 3.25)	7.1×10^{-05}	1.28 (0.84, 1.96)	2.5×10^{-01}	1.10 (0.63, 1.91)	7.3×10^{-01}	3.87 (1.53, 12.93)	1.1×10^{-02}
Interleukin-8	1.77 (1.15, 2.75)	1.0×10^{-02}	1.08 (0.65, 1.79)	7.5×10^{-01}	1.26 (0.70, 2.31)	4.5×10^{-01}	1.63 (0.66, 4.41)	3.0×10^{-01}
Macrophage Inflammatory Protein-1 beta	1.47 (1.02, 2.13)	4.2×10^{-02}	0.92 (0.61, 1.40)	7.1×10^{-01}	1.04 (0.63, 1.73)	8.9×10^{-01}	1.77 (0.72, 4.86)	2.4×10^{-01}
Monocyte Chemotactic Protein 1	2.73 (1.86, 4.11)	5.5×10^{-07}	1.63 (1.05, 2.56)	3.2×10^{-02}	1.47 (0.89, 2.44)	1.3×10^{-01}	3.74 (1.50, 12.58)	1.2×10^{-02}
Placenta Growth Factor	1.67 (1.18, 2.41)	4.7×10^{-03}	1.30 (0.90, 1.92)	1.7×10^{-01}	1.84 (1.12, 3.07)	1.7×10^{-02}	1.50 (0.72, 3.59)	3.1×10^{-01}

Data includes only participants with eGFR > 30 at study day
Odds ratios are per unit of SD of Gaussianised biomarker
CI, confidence interval
Basic clinical covariates: age, sex, diabetes duration, study day eGFR, length of follow-up

ESM Table 4: Associations of each biomarker (considered separately) with having an achieved eGFR < 45, overall and stratified by albuminuria status at study day

	Overall (N=1573, 74 events)				Normo/micro (n=1529, 53 evs)		Macro (n=44, 21 events)	
	Basic covariates		Basic covariates + ACR		Basic covariates		Basic covariates	
Biomarker	OR (95% CI)	p value	OR (95% CI)	p value	OR (95% CI)	p value	OR (95% CI)	p value
**Serum biomarkers**								
Alpha-1-Microglobulin	1.97 (1.54, 2.52)	6.6×10^{-08}	1.47 (1.11, 1.94)	6.5×10^{-03}	1.75 (1.30, 2.34)	1.6×10^{-04}	1.23 (0.53, 3.02)	6.3×10^{-01}
CD27 antigen	3.80 (2.66, 5.55)	9.0×10^{-13}	2.64 (1.80, 3.93)	1.0×10^{-06}	3.02 (2.03, 4.59)	1.0×10^{-07}	3.18 (1.26, 11.29)	3.3×10^{-02}
Clusterin	1.03 (0.72, 1.44)	8.8×10^{-01}	0.98 (0.66, 1.44)	9.2×10^{-01}	1.10 (0.72, 1.64)	6.5×10^{-01}	0.62 (0.24, 1.40)	2.7×10^{-01}
Cystatin-C	1.80 (1.31, 2.50)	4.0×10^{-04}	1.53 (1.08, 2.18)	1.7×10^{-02}	1.99 (1.36, 2.98)	5.8×10^{-04}	0.71 (0.31, 1.50)	3.8×10^{-01}
Kidney Injury Molecule-1	2.77 (2.15, 3.62)	1.3×10^{-14}	2.05 (1.55, 2.75)	7.8×10^{-07}	2.48 (1.86, 3.35)	9.9×10^{-10}	1.52 (0.70, 3.62)	3.0×10^{-01}
Matrix Metalloproteinase-8	1.06 (0.81, 1.37)	6.8×10^{-01}	1.01 (0.77, 1.33)	9.4×10^{-01}	1.10 (0.81, 1.49)	5.4×10^{-01}	0.60 (0.26, 1.21)	1.8×10^{-01}
Syndecan 1	2.46 (1.84, 3.34)	2.7×10^{-09}	1.91 (1.40, 2.62)	4.8×10^{-05}	2.21 (1.60, 3.12)	3.1×10^{-06}	1.11 (0.55, 2.35)	7.6×10^{-01}
Thrombomodulin	1.88 (1.40, 2.56)	4.1×10^{-05}	1.46 (1.06, 2.03)	2.3×10^{-02}	1.57 (1.13, 2.20)	8.3×10^{-03}	1.11 (0.50, 2.68)	8.0×10^{-01}
Tumour Necrosis Factor Receptor 1	4.39 (2.98, 6.64)	4.3×10^{-13}	2.87 (1.90, 4.44)	1.1×10^{-06}	3.23 (2.12, 5.05)	1.1×10^{-07}	1.99 (0.80, 6.25)	1.8×10^{-01}
**Urine biomarkers**								
Amphiregulin	1.24 (0.92, 1.68)	1.6×10^{-01}	1.04 (0.78, 1.39)	8.0×10^{-01}	0.95 (0.67, 1.36)	7.9×10^{-01}	1.51 (0.67, 3.67)	3.3×10^{-01}
EGF/MCP-1 ratio	0.36 (0.26, 0.49)	1.3×10^{-10}	0.53 (0.37, 0.75)	3.6×10^{-04}	0.51 (0.36, 0.72)	1.7×10^{-04}	0.26 (0.07, 0.67)	1.4×10^{-02}
Epidermal Growth Factor	0.88 (0.65, 1.20)	4.3×10^{-01}	0.96 (0.70, 1.33)	8.2×10^{-01}	0.98 (0.68, 1.39)	8.9×10^{-01}	0.89 (0.37, 2.07)	7.8×10^{-01}
Epidermal Growth Factor Receptor	1.43 (1.08, 1.90)	1.3×10^{-02}	1.00 (0.75, 1.34)	9.9×10^{-01}	0.77 (0.54, 1.09)	1.4×10^{-01}	2.40 (1.07, 6.54)	5.2×10^{-02}
Epiregulin	1.47 (1.10, 1.96)	9.2×10^{-03}	1.15 (0.86, 1.55)	3.4×10^{-01}	1.34 (0.96, 1.87)	8.5×10^{-02}	1.50 (0.72, 3.45)	3.0×10^{-01}
Heparin-Binding EGF-Like Growth Factor	1.05 (0.78, 1.40)	7.7×10^{-01}	0.92 (0.68, 1.23)	5.6×10^{-01}	0.84 (0.59, 1.18)	3.1×10^{-01}	1.72 (0.74, 4.38)	2.2×10^{-01}
Interleukin-18	1.57 (1.19, 2.08)	1.7×10^{-03}	1.09 (0.81, 1.48)	5.5×10^{-01}	1.17 (0.84, 1.63)	3.5×10^{-01}	1.35 (0.65, 2.88)	4.2×10^{-01}
Interleukin-4	0.97 (0.72, 1.30)	8.4×10^{-01}	0.86 (0.64, 1.16)	3.3×10^{-01}	0.78 (0.55, 1.11)	1.7×10^{-01}	1.99 (0.89, 5.29)	1.2×10^{-01}
Interleukin-6	1.63 (1.23, 2.17)	7.8×10^{-04}	1.17 (0.88, 1.57)	2.8×10^{-01}	1.17 (0.83, 1.66)	3.6×10^{-01}	2.63 (1.17, 7.33)	3.4×10^{-02}
Interleukin-8	2.05 (1.50, 2.80)	6.6×10^{-06}	1.45 (1.03, 2.03)	3.3×10^{-02}	1.65 (1.15, 2.39)	7.2×10^{-03}	1.99 (0.86, 5.22)	1.3×10^{-01}
Macrophage Inflammatory Protein-1 beta	1.70 (1.31, 2.24)	1.0×10^{-04}	1.24 (0.94, 1.65)	1.4×10^{-01}	1.48 (1.09, 2.01)	1.3×10^{-02}	1.81 (0.82, 4.66)	1.7×10^{-01}
Monocyte Chemotactic Protein 1	2.40 (1.82, 3.21)	1.6×10^{-09}	1.70 (1.25, 2.33)	8.0×10^{-04}	1.80 (1.31, 2.51)	3.4×10^{-04}	3.50 (1.47, 10.67)	1.2×10^{-02}
Placenta Growth Factor	1.35 (1.04, 1.77)	2.6×10^{-02}	1.15 (0.87, 1.52)	3.3×10^{-01}	1.33 (0.98, 1.82)	7.0×10^{-02}	2.02 (0.88, 5.71)	1.3×10^{-01}

Data includes only participants with eGFR > 45 at study day
Odds ratios are per unit of SD of Gaussianised biomarker
CI, confidence interval
Basic clinical covariates: age, sex, diabetes duration, study day eGFR, length of follow-up
